# Supplementary figures and images for: The structural basis of mRNA recognition and binding by yeast pseudouridine synthase PUS1
Source: PLoS One. 2023 Nov 8;18(11):e0291267. doi: 10.1371/journal.pone.0291267 (PMC10631681; doi:10.1371/journal.pone.0291267)

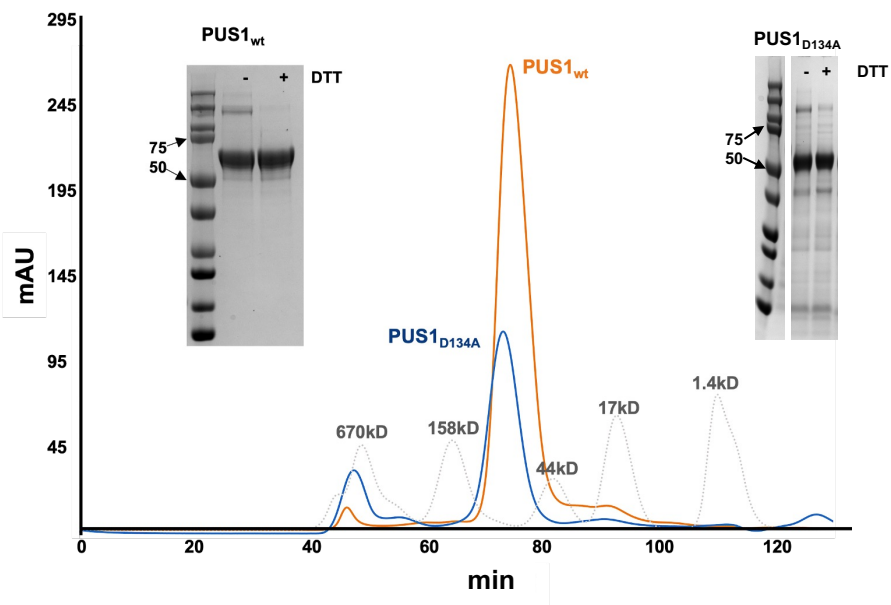

Supplement: S1 Fig — Wildtype PUS1 (PUS1wt, orange) and catalytically inactive PUS1 (PUS1D134A, blue) run as monomers on a HiLoad 16/60 Superdex 200 column. Standards (Bio-Rad Gel Filtration Standards # 1511901) are overlaid in dotted gray and appropriate peaks labeled. (inset) SDS-PAGE of the final purification product of PUS1wt without (-) or with (+) dithiothreitol. (PDF) [file pone.0291267.s001.pdf]

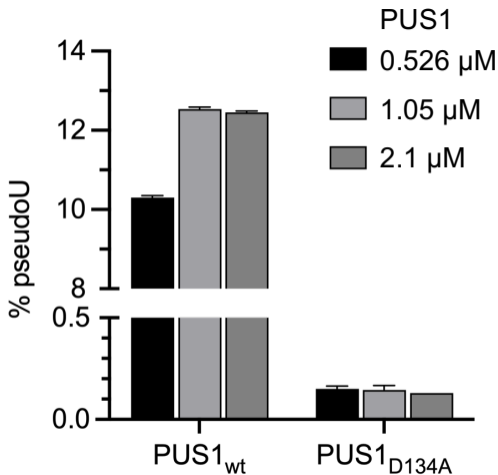

Supplement: S3 Fig — Percent of uridine-to-Ψ conversion as determined by LC-MS/MS of increasing concentrations of PUS1 wildtype (left) and D134A mutant (right) with the RNA oligo substrate R168 after two hours. Data of two replicas are shown. (PDF) [file pone.0291267.s003.pdf]

**a**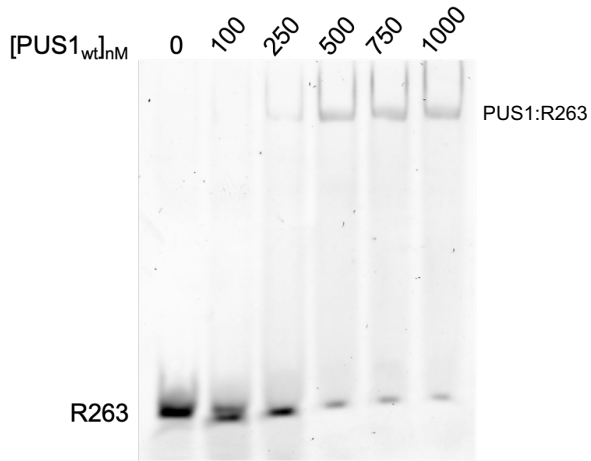**b**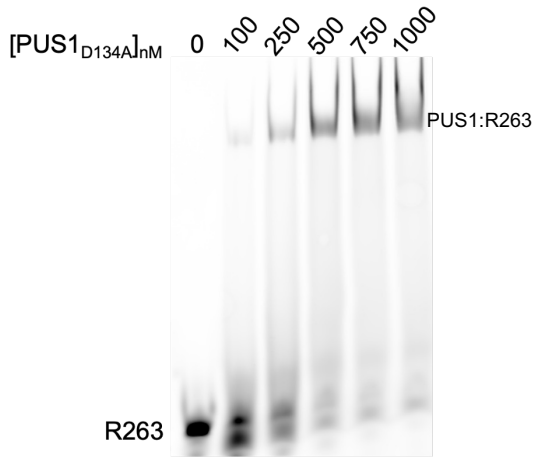

Supplement: S4 Fig — Electrophoretic mobility shift assays of increasing concentrations of wild type PUS1 (left) and PUS1D134A (right) as shown above the gels with 100 nM of RNA oligo R263. Free R263 RNA oligo and the PUS1:RNA complex are indicated. (PDF) [file pone.0291267.s004.pdf]

**a**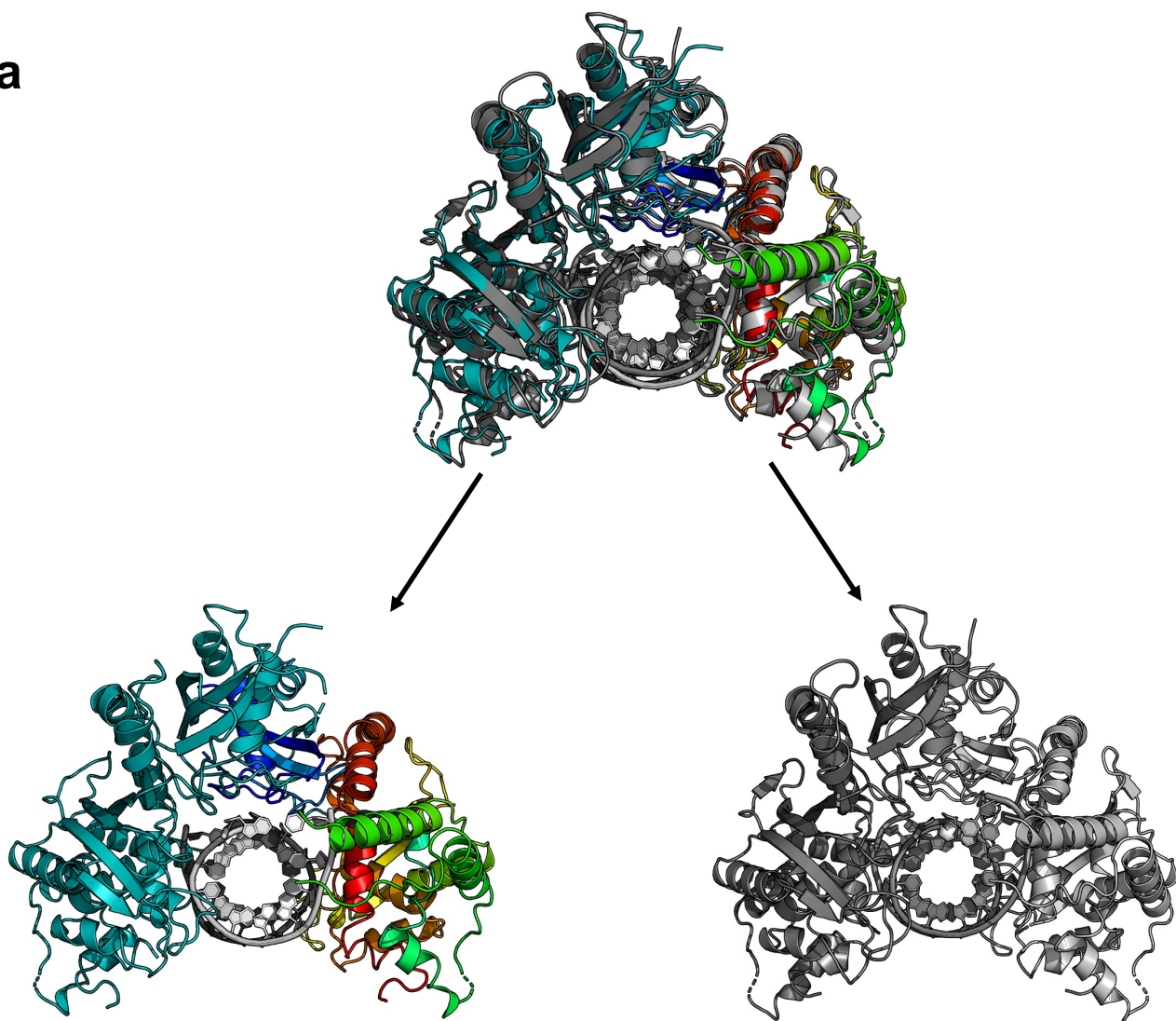**b**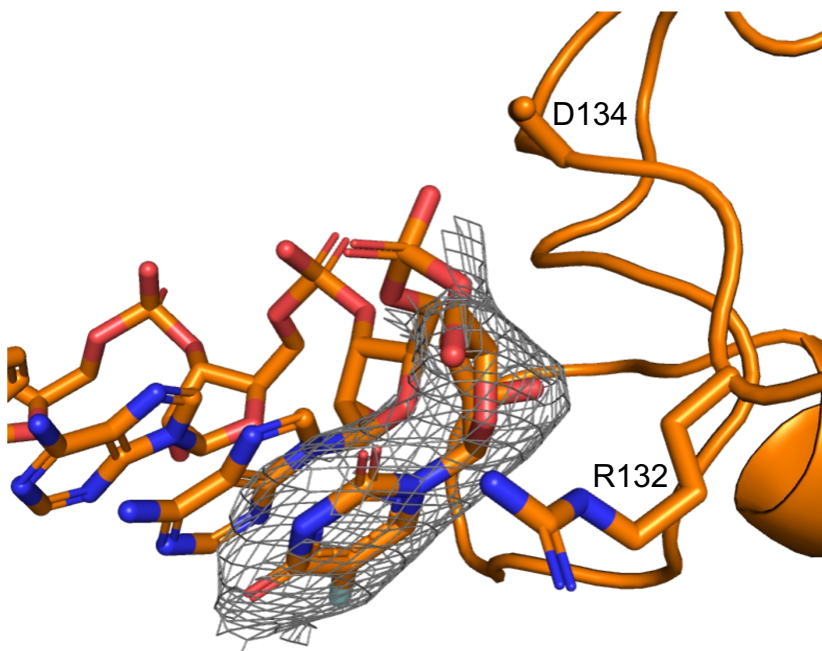

Supplement: S7 Fig — (a) Superposition and side-by-side comparison of PUS1D134A / RNA complex (colored protein backbones) solved in crystallographic space group C2 and wild type PUS1 / RNA complex (grey protein backbones) solved in an unrelated, P6122 space group. In both structures, two monomers of the enzyme are independently bound in a symmetric arrangement to an RNA duplex that is generated via crystallographic 2-fold symmetry. (b) Model and electron density for wild type PUS1 and the region of the RNA containing the 5’ 5-fluorouracil base. A 2Fo-Fc map (gray) is contoured at 1s. In the corresponding refinement, the base is estimated to display greater than 90% occupancy of the unflipped conformation, as modeled and shown. (PDF) [file pone.0291267.s007.pdf]
