# Supplementary material for: The structural basis of mRNA recognition and binding by yeast pseudouridine synthase PUS1
Source: PLoS One. 2023 Nov 8;18(11):e0291267. doi: 10.1371/journal.pone.0291267 (PMC10631681; doi:10.1371/journal.pone.0291267)

a

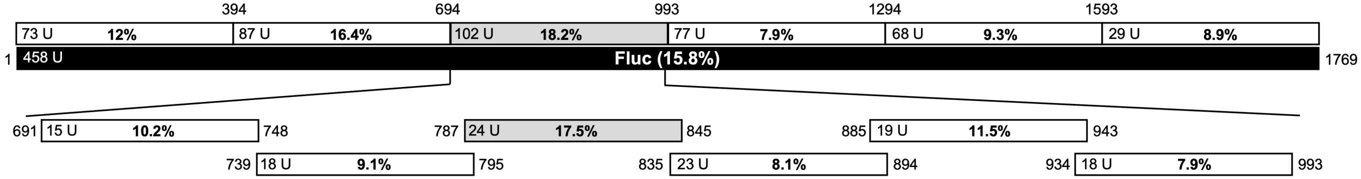

b

RNA	sequence	% Ψ	# uridines	size
IVT	AUUCCGGAUACUGCGAUUUUAAGUGUUGUUCCAUUCCAUCACGGUUUUGGAAUGUUUAC	17.5	24	59
R164	GGGAUUCCGGAUACUGCGAUUUUAAGUGUUG	6.5	11	31
R165	UUCCAUUCCAUCACGGUUUUGGAAUGUUUAC	1.0	13	31
R166	CGAUUUUAAGUGUUGUUCCAUUCCAUCACG	0.3	12	30
R167	UUUUUGGCAAUCAAAUCGGGAUUCCGGAUA	11.3	10	29
R168	GGGUUUUUGGCAAUCAAAUCAUUCCGGAUA	13.1	10	30
R169	AGAGAUCCUAUUUUUGGCAAUCAAAUCGGGAUUCCGGAUA	17.2	12	40
R170	GGGAUUCCGGAUACUGCGAUUUUAAGUGUUGUUCCAUUCC	4.3	15	40
R263	AAAUCGGGAUUCCGGAUA	1.0	4	18

c

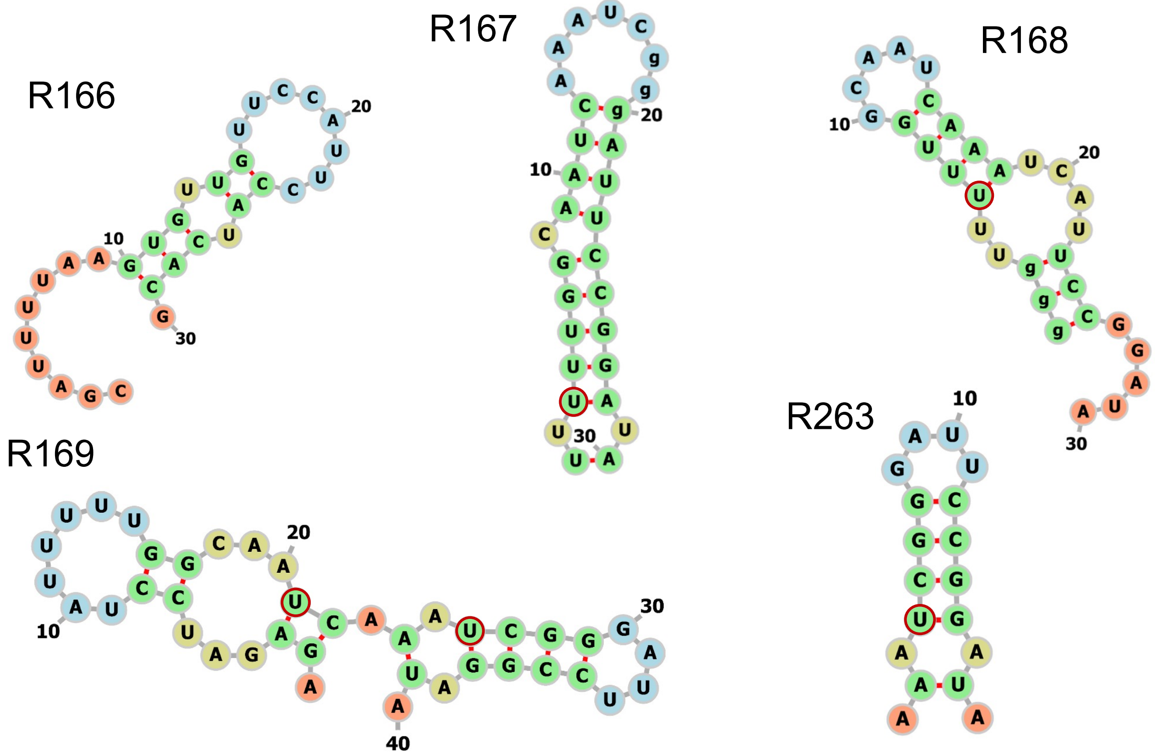

Supplement: S2 Fig — (a) Schematic view of the RNA substrates generated and tested to determine the optimal RNA substrate for crystallography. The black bar represents the full-length Firefly luciferase (Fluc) mRNA sequence. The six bars above Fluc represent ~ 300-mers of the Fluc sequence. The respective start- and end-positions are shown above the segments, the number of total uridines per segment (x U) and the percent of uridine-to-Ψ conversion in a PUS1 reaction after two hours (data from two independent replicas) are shown inside the bars. The bars below Fluc represent ~ 60-mer substrates between Fluc positions 691 and 993. (c) Table showing the sequence of the RNA oligos tested (uridines are highlighted in red), the average percent of uridine-to-Ψ conversion from at least two independent experimental replicates, the number of uridines per RNA oligo, and its respective size in nucleotides. (d) RNA secondary structure predictions of the RNA oligo substrates by MXfold2 [48]. Uridines at the base of a stem-loop structure and thus potential PUS1 targets, are highlighted by a red circle. The nucleotides are colored according to the type of structure that they are in (green: stems (canonical helices), yellow: interior loops, blue: hairpin loops, orange: 5’ and 3’ unpaired region). (PDF) [file pone.0291267.s002.pdf]
