# Supplementary material for: The structural basis of mRNA recognition and binding by yeast pseudouridine synthase PUS1
Source: PLoS One. 2023 Nov 8;18(11):e0291267. doi: 10.1371/journal.pone.0291267 (PMC10631681; doi:10.1371/journal.pone.0291267)
Supplement: S5 Fig — Amino acid residues that interact with the substrate RNA are highlighted by red boxes. (PDF) [file pone.0291267.s005.pdf]

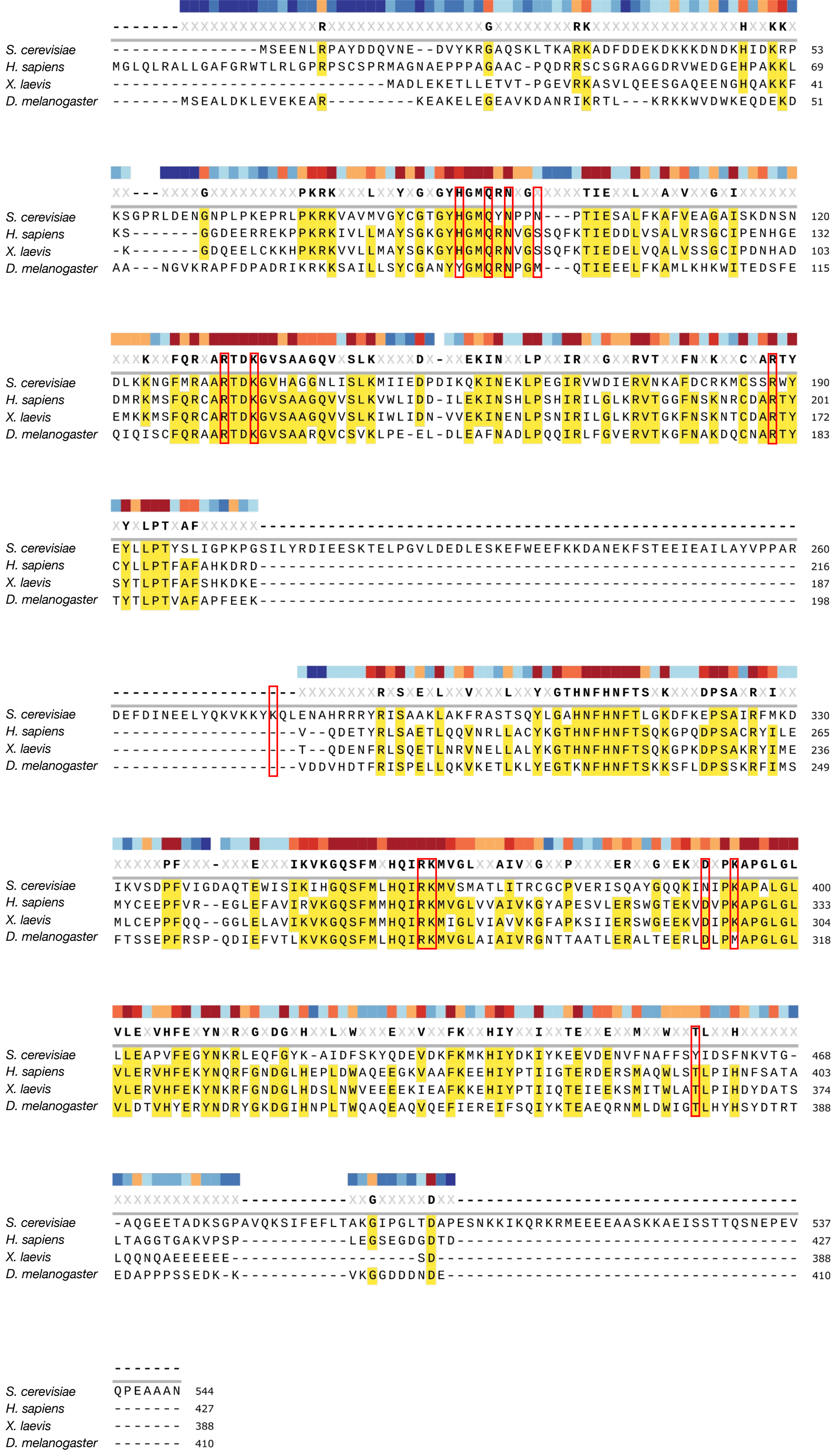

-------XXXXXXXXXXXXXXRXXXXXXXXXXXXXXXXGXXXXXXXXRKXXXXXXXXXXXXXXXHXXKKX
S. cerevisiae ---------------MSEENLRPAYDDQVNE--DVYKRGAQSKLTKARKADFDDEKDKKKDNDKHIDKRP 53
H. sapiens MGLQLRALLGAFGRWTLRLGPRPSCSPRMAGNAEPPPAGAAC-PQDRRSCSGRAGGDRVWEDGEHPAKKL 69
X. laevis ----------------------------MADLEKETLLETVT-PGEVRKASVLQEESGAQEENGHQAKKF 41
D. melanogaster -------MSEALDKLEVEKEAR---------KEAKELEGEAVKDANRIKRTL---KRKKWVDWKEQDEKD 51
XX---XXXXGXXXXXXXXXPKRKXXXLXXYXGXGYHGMQRNXGXXXXXTIEXXLXXAXVXXGXIXXXXXX
S. cerevisiae KSGPRLDENGNPLPKEPRLPKRKVAVMVGYCGTGYHGMQYNPPN---PTIESALFKAFVEAGAISKDNSN 120
H. sapiens KS-------GGDEERREKPPKRKIVLLMAYSGKGYHGMQRNVGSSQFKTIEDDLVSALVRSGCIPENHGE 132
X. laevis -K-------GDQEELCKKHPKRKVVLLMAYSGKGYHGMQRNVGSSQFKTIEDELVQALVSSGCIPDNHAD 103
D. melanogaster AA---NGVKRAPFDPADRIKRKKSAILLSYCGANYYGMQRNPGM---QTIEEELFKAMLKHKWITEDSFE 115
XXXKXXFQRXARTDKGVSAAGQVXSLKXXXXDX-XXEKINXXLPXXIRXXGXXRVTXXFNXKXXCXARTY
S. cerevisiae DLKKNGFMRAARTDKGVHAGGNLISLKMIIEDPDIKQKINEKLPEGIRVWDIERVNKAFDCRKMCSSRWY 190
H. sapiens DMRKMSFQRCARTDKGVSAAGQVVSLKVWLIDD-ILEKINSHLPSHIRILGLKRVTGGFNSKNRCDARTY 201
X. laevis EMKKMSFQRCARTDKGVSAAGQVVSLKIWLIDN-VVEKINENLPSNIRILGLKRVTGKFNSKNTCDARTY 172
D. melanogaster QIQISCFQRAARTDKGVSAARQVCSVKLPE-EL-DLEAFNADLPQQIRLFGVERVTKGFNAKDQCNARTY 183
XYXLPTXAFXXXXXX---
S. cerevisiae EYLLPTYSLIGPKPGSILYRDIEESKTELPGVLDEDLESKEFWEEFKKDANEKFSTEEIEAILAYVPPAR 260
H. sapiens CYLLPTFAFAHKDRD--- 216
X. laevis SYTLPTFAFSHKDKE--- 187
D. melanogaster TYTLPTVAFAPFEEK--- 198
-------------------XXXXXXXXRXSXEXLXXVXXXLXXYXGTHNFHNFTSXKXXXDPSAXRXIXX
S. cerevisiae DEFDINEELYQKVKKYKQLENAHRRRYRISAAKLAKFRASTSQYLGAHNFHNFTLGKDFKEPSAIRFMKD 330
H. sapiens -------------------V--QDETYRLSAETLQQVNRLLACYKGTHNFHNFTSQKGPQDPSACRYILE 265
X. laevis -------------------T--QDENFRLSQETLNRVNELLALYKGTHNFHNFTSQKGPKDPSAKRYIME 236
D. melanogaster -------------------VDDVHDTFRISPELLQKVKETLKLYEGTKNFHNFTSKKSFLDPSSKRFIMS 249
XXXXXPFXXX-XXXEXXXIKVKGQSFMXHQIRKMVGLXXAIVXGXXPXXXXERXXGXEKXDXPKAPGLGL
S. cerevisiae IKVSDPFVIGDAQTEWISIKIHGQSFMLHQIRKMVSMATLITRCGCPVERISQAYGQQKINIPKAPALGL 400
H. sapiens MYCEEPFVR--EGLEFAVIRVKGQSFMMHQIRKMVGLVVAIVKGYAPESVLERSWGTEKVDVPKAPGLGL 333
X. laevis MLCEPPFQQ--GGLELAVIKVKGQSFMMHQIRKMIGLVIAVVKGFAPKSIIERSWGEEKVDIPKAPGLGL 304
D. melanogaster FTSSEPFRSP-QDIEFVTLKVKGQSFMLHQIRKMVGLAIAIVRGNTTAATLERALTEERLDLPMAPGLGL 318
VLEXVHFEXYNXRXGXDGXHXXLXWXXXEXXVXXFKXXHIYXXIXXTEXXEXXMXXWXXTLXXHXXXXXX
S. cerevisiae LLEAPVFEGYNKRLEQFGYK-AIDFSKYQDEVDKFKMKHIYDKIYKEEVDENVFNAFFSYIDSFNKVTG- 468
H. sapiens VLERVHFEKYNQRFGNDGLHEPLDWAQEEGKVAAFKEEHIYPTIIGTERDERSMAQWLSTLPIHNFSATA 403
X. laevis VLERVHFEKYNKRFGNDGLHDSLNWVEEEKIEAFKKEHIYPTIIQTEIEEKSMITWLATLPIHDYDATS 374
D. melanogaster VLDTVHYERYNDRYGKDGIHNPLTWQAQEAQVQEFIEREIFSQIYKTEAEQRNMLDWIGTLHYHSYDTRT 388
XXXXXXXXXXXXX-----------XXGXXXXXDXX-----------------------------------
S. cerevisiae -AQGEETADKSGPAVQKSIFEFLTAKGIPGLTDAPESNKKIKQRKRMEEEEAASKKAEISSTTQSNEPEV 537
H. sapiens LTAGGTGAKVPSP-----------LEGSEGDGDTD----------------------------------- 427
X. laevis LQQNQAEEEEEE-------------------SD------------------------------------- 388
D. melanogaster EDAPPPSSEDK-K-----------VKGGDDDNDE------------------------------------ 410

S. cerevisiae QPEAAAN 544
H. sapiens ------- 427
X. laevis ------- 388
D. melanogaster ------- 410
