# Supplementary material for: The structural basis of mRNA recognition and binding by yeast pseudouridine synthase PUS1
Source: PLoS One. 2023 Nov 8;18(11):e0291267. doi: 10.1371/journal.pone.0291267 (PMC10631681; doi:10.1371/journal.pone.0291267)

a

PUS1 variant	% pseudoU	% activity
$PUS1_{wt}$	15.8	100.0
$PUS1_{H89A}$	11.2	70.8
$PUS1_{R132A}$	2.1	13.3
$PUS1_{R362A}$	3.8	24.3
$PUS1_{K363A}$	8.3	52.9

b

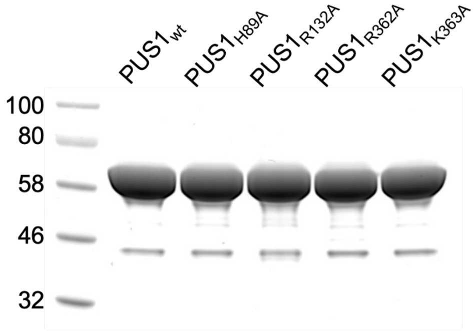

c

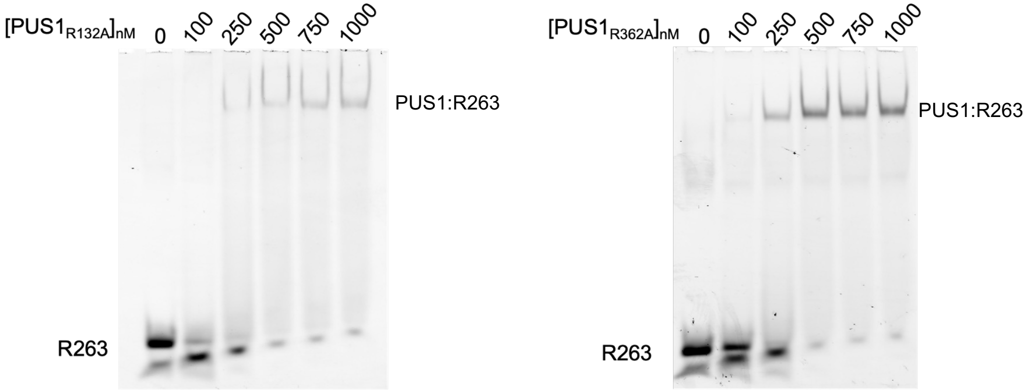

Supplement: S6 Fig — (a) Table showing the average percent of uridine-to-Ψ conversion of the reactions shown in Fig 3C (from two independent replicates), and the percentage of PUS1 activity, normalized by the percent pseudouridylation in the PUS1wt reaction. (b) Protein gel comparing the input amount and purity of the PUS1 variants in the in vitro reactions. (c) Electrophoretic mobility shift assays of the mutant PUS1 enzymes (PUS1R132A left, PUS1R362A right). (PDF) [file pone.0291267.s006.pdf]
