# Supplementary material for: The structural basis of mRNA recognition and binding by yeast pseudouridine synthase PUS1
Source: PLoS One. 2023 Nov 8;18(11):e0291267. doi: 10.1371/journal.pone.0291267 (PMC10631681; doi:10.1371/journal.pone.0291267)
Supplement: S8 Fig — The conserved catalytic aspartate at position 134 in PUS1 and position 60 in TruA is indicated by a yellow box. RNA-interacting residues are highlighted in green (PUS1) and magenta (TruA) (top panel). Distribution of RNA-contacting residues in the protein-subunit interface for PUS1 (left) and TruA (right; bottom panel). (PDF) [file pone.0291267.s008.pdf]

```
scPUS1      MSEENLRPAYDDQVNEDVYKRGAQSKLTKARKADFDDEKDKKKDNDKHIDKRPKSGPRLD      60
ecTruA      ------------------------------------------------------------      0


scPUS1      ENGNPLPKEPRLPKRKVAVMVGYCGTGYHGMQYNPPNPTIESALFKAFVEAGAISKDNSN      120
ecTruA      -----MSDQQQPPVYKIALGIEYDGSKYYGWQRQNEVRSVQEKLEKALSQVA--------      47
                 : .: : *  *:*: : * *: *:* * :    :::. * **: :..

scPUS1      DLKKNGFMRAARTDKGVHAGGNLISLKMII--EDPDIKQKINEKLPEGIRVWDIERVNKA      178
ecTruA      -NEPITVFCAGRTDAGVHGTGQVVHFETTALRKDAAWTLGVNANLPGDIAVRWVKTVPDD      106
              :   .: *.*** ***. *::: ::     :*   .  :* :** .* *  :: * .

scPUS1      FDCRKMCSSRWYEYLLPTYSLIGPKPGSILYRDIEESKTELPGVLDEDLESKEFWEEFKK      238
ecTruA      FHARFSATARRYRYIIYNHRLRP-------------------AVLSKGV--THFYEPLDA      145
            *..*  .::* *.*:: .: *                     .**.:.:  ..*:* :.

scPUS1      DANEKFSTEEIEAILAYVPPARDEFDINEELYQKVKKYKQLENAHRRRYRISAAKLAKFR      298
ecTruA      ERM--------------------------------------------------------H      149
            :                                                          :

scPUS1      ASTSQYLGAHNFHNFTLGKDF-KEPSAIRFMKDIKVSDPFVIGDAQTEWISIKIHGQSFM      357
ecTruA      RAAQCLLGENDFTSFRAVQCQSRTPWRNVMHINVTRHGPY---------VVVDIKANAFV      200
             ::.  ** ::* .*   :   : *    :  ::.  .*:         : :.*:.::*:

scPUS1      LHQIRKMVSMATLITRCGCPVERISQAY-----------------------GQQKINIPK      394
ecTruA      HHMVRNIVGSLMEVGAHNQPESWIAELLAAKDRTLAAATAKAEGLYLVAVDYPDRYDLPK      260
             * :*::*.    :   . * . *::                           :: ::**

scPUS1      APALGLLLEAPVFEGYNKRLEQFGYKAIDFSKYQDEVDKFKMKHIYDKIYKEEVDENVFN      454
ecTruA      PPMGPLFLAD--------------------------------------------------      270
             *   *:*

scPUS1      AFFSYIDSFNKVTGAQGEETADKSGPAVQKSIFEFLTAKGIPGLTDAPESNKKIKQRKRM      514
ecTruA      ------------------------------------------------------------      270


scPUS1      EEEEAASKKAEISSTTQSNEPEVQPEAAAN 544
ecTruA      ------------------------------ 270
```

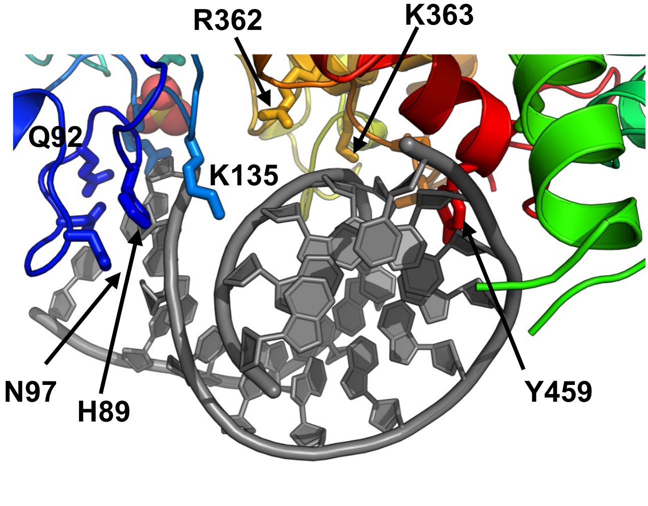

PUS1

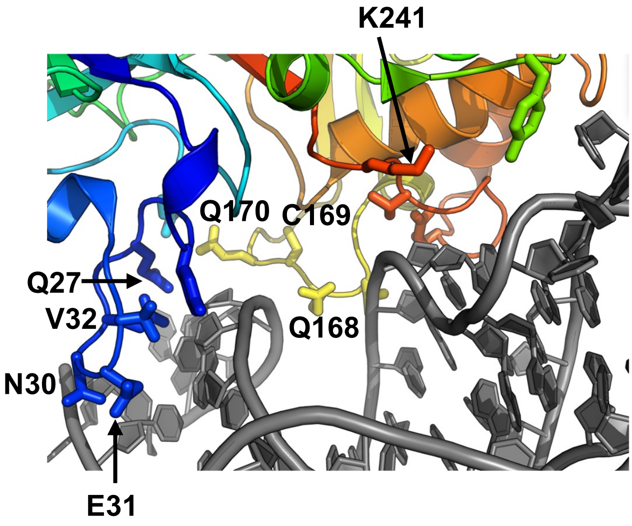

TruA
