# Supplementary material for: The structural basis of mRNA recognition and binding by yeast pseudouridine synthase PUS1
Source: PLoS One. 2023 Nov 8;18(11):e0291267. doi: 10.1371/journal.pone.0291267 (PMC10631681; doi:10.1371/journal.pone.0291267)
Supplement: S1 File — (PDF) [file pone.0291267.s009.pdf]

# Supporting Information

## Supporting materials and methods

### Electrophoretic mobility shift assays

100 nM (10 pmol) to 1 µM (100 pmol) of the wildtype and the mutant PUS1 enzymes were incubated with 100 nM (10 pmol) of the synthetic RNA oligo R263 in a 100 µl reaction under PUS1 activity assay conditions (see Materials and Methods) for 90 min at 30°C. Immediately before loading onto the gel, 18 µl of each reaction were transferred to a fresh tube containing 2 µl native PAGE loading dye (0.05% xylene cyanol, 50% glycerol). 18 µl of each sample were then loaded on a 6% Novex™ TBE gel that was running at 150 V at 4°C. After 30 min, the gel was stopped and visualized using the Cy2 channel of an Amersham Typhoon Laser Scanner after staining with SYBR Gold (1/20,000 dilution, Thermo Fisher Scientific # S11494) for 10 min.

### Determination of Ψ sites by oligonucleotide UHPLC-MS/MS

3.9 pmol of PUS1-modified and unmodified R397, R398, and R444 oligonucleotides (sequences are listed in S3 Table) were digested with 1 µL of a 1:50 dilution of RNaseT1 (Thermo Fisher Scientific) in a 30 µL reaction in 1 x NEB buffer r1.1 (10 mM Bis-Tris-Propane-HCl, 10 mM $MgCl_2$, 100 µg/ml Recombinant Albumin, pH 7.0) at 37 °C for 30 minutes. RNaseT1 cleavage products were spin filtered at 13,400 rpm for 5 minutes utilizing Ultrafree MC-GV 0.22 µm spin filters (Millipore).

Filtered oligonucleotides were analyzed by UHPLC-MS/MS. UHPLC analysis was performed on a Vanquish Horizon UHPLC (Thermo Fisher Scientific) utilizing an ACQUITY Premier Oligonucleotide C18 Column (Waters) (2.1 x 100 mm, 1.7 µm) with a 23-minute 7% - 35% gradient of solvent A (1% hexafluoroisopropanol (HFIP), 0.1% N,N-diisopropylethylamine (DIEA), 1 µM EDTA) and solvent B (90% Methanol, 10% water, 0.075% HFIP, 0.0375% DIEA, 1 µM EDTA) at a flow rate of 400 µL/min (at 60°C). MS/MS analysis was performed on an Eclipse Fusion Orbitrap Mass Spectrometer (Thermo Fisher Scientific) in data-dependent acquisition mode at a resolution of 60,000 using high energy collision dissociation (HCD) with a stepped normalized collision energy of 22, 24 and 26%.

MS/MS-based oligonucleotide sequencing data were searched utilizing the Nucleic Acid Search Engine (NASE) tool in OpenMS [1] with a 5% false discovery rate and RNase T1 digests of corresponding R397 or R398 oligonucleotide sequences. Pseudo-uridylated oligonucleotides were detected by a procedure adapted from Yamauchi *et al*. [2]. Briefly, NASE annotated MS/MS spectra containing a 207.04 m/z doubly dehydrated $\Psi$ nucleoside signature ion and a 211.00 m/z ribose phosphate ion with a mass accuracy tolerance of +/- 0.005 m/z were identified and summed.

## Supporting Information References

1. Wein S, Andrews B, Sachsenberg T, Santos-Rosa H, Kohlbacher O, Kouzarides T, et al. A computational platform for high-throughput analysis of RNA sequences and modifications by mass spectrometry. Nat Commun. 2020;11(1):926. Epub 2020/02/19.

doi: 10.1038/s41467-020-14665-7. PubMed PMID: 32066737; PubMed Central PMCID: PMCPMC7026122.

2. Yamauchi Y, Nobe Y, Izumikawa K, Higo D, Yamagishi Y, Takahashi N, et al. A mass spectrometry-based method for direct determination of pseudouridine in RNA. Nucleic Acids Res. 2016;44(6):e59. Epub 2015/12/18. doi: 10.1093/nar/gkv1462. PubMed PMID: 26673725; PubMed Central PMCID: PMCPMC4824092.
