# Supplementary material for: The structural basis of mRNA recognition and binding by yeast pseudouridine synthase PUS1
Source: PLoS One. 2023 Nov 8;18(11):e0291267. doi: 10.1371/journal.pone.0291267 (PMC10631681; doi:10.1371/journal.pone.0291267)
Supplement: S1 Table — (PDF) [file pone.0291267.s010.pdf]

>6HisPUS1
tggcgaatgggacgcgccctgtagcggcgcattaagcgcggcgggtgtggtggttacgcgcagcgtgaccgctacacttgccagcgccctagcgc
ccgctcctttcgctttcttcccttcctttctcgccacgttcgccggctttccccgtcaagctctaaatcgggggctccctttagggttccgatttagtgctttacg
gcacctcgaccccaaaaaacttgattagggtgatggttcacgtagtgggccatcgccctgatagacggtttttcgccctttgacgttggagtccacgttct
ttaatagtggactcttgttccaaactggaacaacactcaaccctatctcggtctattcttttgatttataagggattttgccgatttcggcctattggttaaaaa
atgagctgatttaacaaaaatttaacgcgaattttaacaaaatattaacgtttacaatttcaggtggcacttttcggggaaatgtgcgcggaacccctattt
gtttatttttctaaatacattcaaatatgtatccgctcatgaattaattcttagaaaaactcatcgagcatcaaatgaaactgcaatttattcatatcaggatta
tcaataccatatttttgaaaaagccgtttctgtaatgaaggagaaaactcaccgaggcagttccataggatggcaagatcctggtatcggtctgcgattc
cgactcgtccaacatcaatacaacctattaatttcccctcgtcaaaaataaggttatcaagtgagaaatcaccatgagtgacgactgaatccggtgag
aatggcaaaagtttatgcatttctttccagacttgttcaacaggccagccattacgctcgtcatcaaaatcactcgcatcaaccaaaccgttattcattcgt
gattgcgcctgagcgagacgaaatacgcgatcgctgttaaaaggacaattacaaacaggaatcgaatgcaaccggcgcaggaacactgccagc
gcatcaacaatattttcacctgaatcaggatattcttctaatacctggaatgctgttttcccggggatcgcagtggtgagtaaccatgcatcatcaggagt
acggataaaatgcttgatggtcggaagaggcataaattccgtcagccagtttagtctgaccatctcatctgtaacatcattggcaacgctacctttgcca
tgtttcagaaacaactctggcgcatcgggcttcccatacaatcgatagattgtcgcacctgattgcccgacattatcgcgagcccatttatacccatata
aatcagcatccatgttggaatttaatcgcggcctagagcaagacgtttcccgttgaatatggctcataacaccccttgtattactgtttatgtaagcagac
agttttattgttcatgaccaaaatcccttaacgtgagttttcgttccactgagcgtcagaccccgtagaaaagatcaaaggatcttcttgagatcctttttttct
gcgcgtaatctgctgcttgcaaacaaaaaaaccaccgctaccagcggtggtttgtttgccggatcaagagctaccaactctttttccgaaggtaactgg
cttcagcagagcgcagataccaaatactgtccttctagtgtagccgtagttaggccaccacttcaagaactctgtagcaccgcctacatacctcgctct
gctaatcctgttaccagtggctgctgccagtggcgataagtcgtgtcttaccgggttggactcaagacgatagttaccggataaggcgcagcggtcgg
gctgaacggggggttcgtgcacacagcccagcttggagcgaacgacctacaccgaactgagatacctacagcgtgagctatgagaaagcgcca
cgcttcccgaagggagaaaggcggacaggtatccggtaagcggcagggtcggaacaggagagcgcacgagggagcttccagggggaaacg
cctggtatctttatagtcctgtcgggtttcgccacctctgacttgagcgtcgatttttgtgatgctcgtcaggggggcggagcctatggaaaaacgccagc
aacgcggcctttttacggttcctggccttttgctggccttttgctcacatgttctttcctgcgttatcccctgattctgtggataaccgtattaccgcctttgagtga
gctgataccgctcgccgcagccgaacgaccgagcgcagcgagtcagtgagcgaggaagcggaagagcgcctgatgcggtattttctccttacgc
atctgtgcggtatttcacaccgcatatatggtgcactctcagtacaatctgctctgatgccgcatagttaagccagtatacactccgctatcgctacgtga
ctgggtcatggctgcgccccgacacccgccaacacccgctgacgcgccctgacgggcttgtctgctcccggcatccgcttacagacaagctgtgac
cgtctccgggagctgcatgtgtcagaggttttcaccgtcatcaccgaaacgcgcgaggcagctgcggtaaagctcatcagcgtggtcgtgaagcgat
tcacagatgtctgcctgttcatccgcgtccagctcgttgagtttctccagaagcgttaatgtctggcttctgataaagcgggccatgttaagggcggttttttc
ctgtttggtcactgatgcctccgtgtaagggggatttctgttcatgggggtaatgataccgatgaaacgagagaggatgctcacgatacgggttactgat
gatgaacatgcccggttactggaacgttgtgagggtaaacaactggcggtatggatgcggcgggaccagagaaaaatcactcagggtcaatgcca
gcgcttcgttaatacagatgtaggtgttccacagggtagccagcagcatcctgcgatgcagatccggaacataatggtgcagggcgctgacttccgc
gtttccagactttacgaaacacggaaaccgaagaccattcatgttgttgctcaggtcgcagacgttttgcagcagcagtcgcttcacgttcgctcgcgta
tcggtgattcattctgctaaccagtaaggcaaccccgccagcctagccgggtcctcaacgacaggagcacgatcatgcgcacccgtggggccgcc
atgccggcgataatggcctgcttctcgccgaaacgtttggtggcgggaccagtgacgaaggcttgagcgagggcgtgcaagattccgaataccgc
aagcgacaggccgatcatcgtcgcgctccagcgaaagcggtcctcgccgaaaatgacccagagcgctgccggcacctgtcctacgagttgcatg
ataaagaagacagtcataagtgcggcgacgatagtcatgccccgcgcccaccggaaggagctgactgggttgaaggctctcaagggcatcggtc
gagatcccggtgcctaatgagtgagctaacttacattaattgcgttgcgctcactgcccgctttccagtcgggaaacctgtcgtgccagctgcattaatg
aatcggccaacgcgcggggagaggcggtttgcgtattgggcgccagggtggtttttcttttcaccagtgagacgggcaacagctgattgcccttcacc
gcctggccctgagagagttgcagcaagcggtccacgctggtttgccccagcaggcgaaaatcctgtttgatggtggttaacggcgggatataacatg
agctgtcttcggtatcgtcgtatcccactaccgagatatccgcaccaacgcgcagcccggactcggtaatggcgcgcattgcgcccagcgccatctg
atcgttggcaaccagcatcgcagtgggaacgatgccctcattcagcatttgcatggtttgttgaaaaccggacatggcactccagtcgccttcccgttcc
gctatcggctgaatttgattgcgagtgagatatttatgccagccagccagacgcagacgcgccgagacagaacttaatgggcccgctaacagcgcg
atttgctggtgacccaatgcgaccagatgctccacgcccagtcgcgtaccgtcttcatgggagaaaataatactgttgatgggtgtctggtcagagac
atcaagaaataacgccggaacattagtgcaggcagcttccacagcaatggcatcctggtcatccagcggatagttaatgatcagcccactgacgcg
ttgcgcgagaagattgtgcaccgccgctttacaggcttcgacgccgcttcgttctaccatcgacaccaccacgctggcacccagttgatcggcgcga
gatttaatcgccgcgacaatttgcgacggcgcgtgcagggccagactggaggtggcaacgccaatcagcaacgactgtttgcccgccagttgttgtg
ccacgcggttgggaatgtaattcagctccgccatcgccgcttccactttttcccgcgttttcgcagaaacgtggctggcctggttcaccacgcgggaaa
cggtctgataagagacaccggcatactctgcgacatcgtataacgttactggtttcacattcaccaccctgaattgactctcttccgggcgctatcatgcc
ataccgcgaaaggttttgcgccattcgatggtgtccgggatctcgacgctctcccttatgcgactcctgcattaggaagcagcccagtagtaggttgag
gccgttgagcaccgccgccgcaaggaatggtgcatgcaaggagatggcgcccaacagtcccccggccacggggcctgccaccatacccacgc
cgaaacaagcgctcatgagcccgaagtggcgagcccgatcttccccatcggtgatgtcggcgatataggcgccagcaaccgcacctgtggcgcc

ggtgatgccggccacgatgcgtccggcgtagaggatcgagatctcgatcccgcgaaattaatacgactcactataggggaattgtgagcggataac
aattcccctctagaaataattttgtttaactttaagaaggagatataccatgggcagcagccatcatcatcatcatcacagcagcggcctggtgccgcg
cggcagccatatgagcgaggaaaacctgcgtccggcgtatgacgatcaggttaacgaagacgtgtacaaacgtggtgcgcaaagcaagctgac
caaagcgcgtaaggcggacttcgacgatgagaaagataagaaaaaggacaacgataagcacatcgacaaacgtccgaagagcggtccgcgt
ctggatgaaaacggtaacccgctgccgaaggagccgcgtctgccgaagcgtaaggtggcggttatggtgggttactgcggtaccggctatcacgg
catgcaatacaacccgccgaacccgaccatcgaaagcgcgctgttcaaagcgtttgttgaggcgggtgcgattagcaaggacaacagcaacgat
ctgaaaaagaacggtttcatgcgtgcggcgcgtaccgacaaaggcgtgcatgcgggtggcaacctgatcagcctgaagatgatcattgaagaccc
ggatattaaacagaagatcaacgaaaaactgccggagggcattcgtgtttgggacatcgaacgtgtgaacaaagcgtttgattgccgtaagatgtgc
agcagccgttggtacgagtatctgctgccgacctatagcctgatcggtccgaaaccgggcagcattctgtaccgtgacatcgaggaaagcaagacc
gagctgccgggtgttctggacgaggatctggaaagcaaagagttctgggaggaatttaaaaaggatgcgaacgaaaagttcagcaccgaggaaa
ttgaagcgatcctggcgtatgttccgccggcgcgtgacgaatttgatatcaacgaggaactgtatcagaaagtgaaaaagtacaagcaactggaga
acgcgcaccgtcgtcgttaccgtattagcgcggcgaaactggcgaagttccgtgcgagcaccagccagtacctgggtgcgcacaacttccacaact
ttaccctgggcaaagacttcaaggaaccgagcgcgattcgttttatgaaagacatcaaggttagcgatccgttcgtgatcggtgatgcgcaaaccga
gtggatcagcattaaaatccacggccagagctttatgctgcaccaaattcgtaagatggttagcatggcgaccctgatcacccgttgcggttgcccggt
ggaacgtattagccaggcgtatggccagcaaaaaattaacattccgaaagcgccggcgctgggtctgctgctggaagcgccggttttcgagggtta
caacaagcgtctggaacagttcggctataaagcgatcgactttagcaagtaccaagacgaggtggataagttcaagatgaagcacatctacgaca
agatctacaaggaagaggtggatgagaacgtgttcaacgcgttctttagctacattgacagctttaacaaagttaccggtgcgcagggcgaggaaa
ccgcggataaaagcggcccggcggtgcaaaagagcattttcgaatttctgaccgcgaaaggtattccgggcctgaccgatgcgccggagagcaa
caaaaagatcaaacagcgtaagcgtatggaggaagaggaagcggcgagcaaaaaggcggaaattagcagcaccacccagagcaacgagc
cggaagtgcaaccggaggcggcggcgaactaactcgagcaccaccaccaccaccactgagatccggctgctaacaaagcccgaaaggaagc
tgagttggctgctgccaccgctgagcaataactagcataaccccttggggcctctaaacgggtcttgaggggttttttgctgaaaggaggaactatatc
cggat
