# Supplementary material for: The structural basis of mRNA recognition and binding by yeast pseudouridine synthase PUS1
Source: PLoS One. 2023 Nov 8;18(11):e0291267. doi: 10.1371/journal.pone.0291267 (PMC10631681; doi:10.1371/journal.pone.0291267)
Supplement: S2 Table — (PDF) [file pone.0291267.s011.pdf]

>Fluc_mRNA
GGGUCUAGAAAUAAUUUUGUUUAACUUUAAGAAGGAGAUAUAACCAUGAAAAUCGAAGAAGGUAA
AGGUCACCAUCACCAUCACCACGGAUCCAUGGAAGACGCCAAAAACAUAAAGAAAGGCCCGGCGC
CAUUCUAUCCUCUAGAGGAUGGAACCGCUGGAGAGCAACUGCAUAAGGCUAUGAAGAGAUACGC
CCUGGUUCCUGGAACAAUUGCUUUUACAGAUGCACAUAUCGAGGUGAACAUCACGUACGCGGAA
UACUUCGAAAUGUCCGUUCGGUUGGCAGAAGCUAUGAAACGAUAUGGGCUGAAUACAAAUCACAG
AAUCGUCGUAUGCAGUGAAAACUCUCUUCAAUUCUUUAUGCCGGUGUUGGGCGCGUUAUUUAUC
GGAGUUGCAGUUGCGCCCGCGAACGACAUUUAUAAUGAACGUGAAUUGCUCAACAGUAUGAACA
UUUCGCAGCCUACCGUAGUGUUUGUUUCCAAAAAGGGGUUGCAAAAAAUUUUGAACGUGCAAAAA
AAAUUACCAAUAAUCCAGAAAAUUAUUAUCAUGGAUUCUAAAACGGAUUACCAGGGAUUUCAGUC
GAUGUACACGUUCGUCACAUCUCAUCUACCUCCCGGUUUUAAUGAAUACGAUUUUGUACCAGAG
UCCUUUGAUCGUGACAAAACAAUUGCACUGAUAAUGAAUUCCUCUGGAUCUACUGGGUUACCUAA
GGGUGUGGCCCUUCCGCAUAGAACUGCCUGCGUCAGAUUCUCGCAUGCCAGAGAUCCUAUUUUU
GGCAAUCAAAUCGGGAUUCCGGAUACUGCGAUUUUAAGUGUUGUUCCAUUCCAUCACGGUUUUG
GAAUGUUUACUACACUCGGAUAUUUGAUAUGUGGAUUUCGAGUCGUCUUAAUGUAUAGAUUUGA
AGAAGAGCUGUUUUUACGAUCCCUUCAGGAUUACAAAAUUCAAAGUGCGUUGCUAGUACCAACCC
UAUUUUCAUUCUUCGCCAAAAGCACUCUGAUUGACAAAUACGAUUUAUCUAAUUUACACGAAAUU
GCUUCUGGGGGCGCACCUCUUUCGAAAGAAGUCGGGGAAGCGGUUGCAAAACGCUUCCAUCUUC
CAGGGAUACGACAAGGAUAUGGGCUCACUGAGACUACAUCAGCUAUUCUGAUUACACCCGAGGG
GGAUGAUAAACCGGGCGCGGUCGGUAAAGUUGUUCCAUUUUUUGAAGCGAAGGUUGUGGAUCUG
GAUACCGGGAAAACGCUGGGCGUUAAUCAGAGAGGCGAAUUAUGUGUCAGAGGACCUAUGAUUA
UGUCCGGUUAUGUAAACAAUCCGGAAGCGACCAACGCCUUGAUUGACAAGGAUGGAUGGCUACA
UUCUGGAGACAUAGCUUACUGGGACGAAGACGAACACUUCUUCAUAGUUGACCGCUUGAAGUCU
UUAAUUAAAUACAAAGGAUAUCAGGUGGCCCCCGCUGAAUUGGAAUCGAUAUUGUUACAACACCC
CAACAUCUUCGACGCGGGCGUGGCAGGUCUUCCCGACGAUGACGCCGGUGAACUUCCCGCCGC
CGUUGUUGUUUUGGAGCACGGAAAGACGAUGACGGAAAAAGAGAUCGUGGAUUACGUCGCCAGU
CAAGUAACAACCGCGAAAAAGUUGCGCGGAGGAGUUGUGUUUGUGGACGAAGUACCGAAAGGUC
UUACCGGAAAACUCGACGCAAGAAAAAUCAGAGAGAUCCUCAUAAAGGCCAAGAAGGGCGGAAAG
UCCAAACUCGAGUAAGGUUAACCUGCAGGAGG
