# Supplementary material for: The structural basis of mRNA recognition and binding by yeast pseudouridine synthase PUS1
Source: PLoS One. 2023 Nov 8;18(11):e0291267. doi: 10.1371/journal.pone.0291267 (PMC10631681; doi:10.1371/journal.pone.0291267)
Supplement: S3 Table — (PDF) [file pone.0291267.s012.pdf]

name	sequence
IVT	AUUCCGGAUACUGCGAUUUUAAGUGUUGUUCCAUUCCAUCACGGUUUUGGAAUGUUUAC
R164	GGGAUUCCGGAUACUGCGAUUUUAAGUGUUG
R165	UUCCAUUCCAUCACGGUUUUGGAAUGUUUAC
R166	CGAUUUUAAGUGUUGUUCCAUUCCAUCACG
R167	UUUUUGGCAAUCAAAUCGGGAUUCCGGAUA
R168	GGGUUUUUGGCAAUCAAAUCAUUCCGGAUA
R169	AGAGAUCCUAUUUUUGGCAAUCAAAUCGGGAUUCCGGAUA
R194	AGAGAACCGAUUUUUGGCAAACAAAGCGGGAGCCCGGACA
R195	AGAGAACCGAACCGAGGCAAGCAAAUCGGGAUUCCGGAUA
R263	AAAUCGGGAUUCCGGAUA
R340	/i5F-U/AAUCGGGAUUCCGGAUA
R397	GCUAUAAAUCGGGAUUCCAACGAAUCCCAGGAUUUACCAG
R398	GCUAUAAAUCGGGAUUCCAACGAAUCCCAGGAUUUACCAUAUUCUGGCUGUGUAUCCACAGACAGAACAA
R444	GCUAAAAAUCGGGAUUCCAACGAAUCCCAGGAUUUUCCAG
